# Augmenting the Effectiveness of CAR-T Cells by Enhanced Self-Delivery of PD-1-Neutralizing scFv

**DOI:** 10.3389/fcell.2020.00803

**Published:** 2020-08-18

**Authors:** Yu Ping, Feng Li, Shufeng Nan, Daiqun Zhang, Xiaojuan Shi, Jiqi Shan, Yi Zhang

**Affiliations:** ^1^Biotherapy Center, The First Affiliated Hospital of Zhengzhou University, Zhengzhou, China; ^2^Cancer Center, The First Affiliated Hospital of Zhengzhou University, Zhengzhou, China; ^3^Henan Key Laboratory for Tumor Immunology and Biotherapy, Zhengzhou, China; ^4^School of Life Sciences, Zhengzhou University, Zhengzhou, China

**Keywords:** chimeric antigen receptor T cell, anti-PD-1, single-chain variable fragment, tumor microenvironment, T cell function

## Abstract

Chimeric antigen receptor T (CAR-T) cell therapy is not satisfying in solid tumors. PD-1-mediated suppression greatly hinders CAR-T cells in the microenvironment. It has been shown that PD-1 blockade improves the effectiveness of CAR-T cells. Herein, we designed CAR-T cells than could secret α-PD-1 scFv by themselves. To obtain optimal secretions of scFv, we screened several signal peptides. And the segment from human increased the extracellular production of PD-1-neutralizing proteins. The secreted neutralizing scFv efficiently blocked PD-1 and enhanced T cell activation when PD-L1 was present. Further analysis showed that CAR-T cells themselves could secret α-PD-1 scFv with bioactivity. In contrast to the prototype, the scFv-producing CAR-T cells demonstrated decreased PD-1 but increases expansion and toxicity against solid tumor cells. In the subcutaneous and orthotopic xenograft models, the self-delivered α-PD-1 scFv increased CAR-T cell functionalities and tumor-suppressions. Our work suggested that engineering T cells to co-express antigen-responsive receptors and checkpoint inhibitors is effective to optimize CAR-T cell therapy for solid tumors.

## Introduction

Adoptive transfer therapy with chimeric antigen receptor T (CAR-T) cells has made great progresses in malignant diseases (Minn et al., 2019; Tian et al., 2020). CAR-T cells targeting CD19, CD20, and CD22 have shown promises in treating hematological malignancies in clinical trials (Grupp et al., 2013; Zhang et al., 2016; Fry et al., 2018; Li D. et al., 2019; Li Y. et al., 2019). Nevertheless, CAR-T cells demonstrate very limited benefits to date in solid tumors (Li et al., 2018; Martinez and Moon, 2019; Yang et al., 2019; Zhang et al., 2020). One important issue constraining the effectiveness of CAR-T cells is the inhibitory impacts of immune checkpoints in the microenvironment of solid tumors (Ma et al., 2019; Shi et al., 2019). Hence, unleashing the inhibition would augment CAR-T cell therapy.

Immune checkpoint blockade therapies, especially anti-programmed death-1 (PD-1) antibodies exhibit encouraging responses in clinic settings (Borghaei et al., 2015; Cheng et al., 2018). Anti-PD-1 (α-PD-1) therapy can reduce the inhibitory effects on T cells in solid tumors, hence increasing the proliferation, cytotoxicity and tumoral accumulation of T cells (Huang et al., 2019; Tunger et al., 2019). Similarly with the natural T cells, CAR-T cells infiltrated in malignant tissues are vigorously suppressed by PD-1/PD-L1 signal (Cherkassky et al., 2016). Several studies have reported that CAR-T cell therapy is improved by PD-1/PD-L1 blockade in several solid tumors (John et al., 2013; Gargett et al., 2016; Gulati et al., 2018). Therefore, it has attracted lots of attentions to combine anti-PD-1 with CAR-T cells to improve the effectiveness of the engineered T cell therapy in solid tumor. Nevertheless, the systemic infusion of PD-1-blocking antibody raises many concerns including non-specific T cell activation, low concentrations of antibody in tumors, and elevated clinical costs.

To overcome the inhibitory effects of PD-1, synergistically administrating anti-PD-1 antibody or deleting PD-1 with CRISPR/cas9 have been extensively studied in CAR-T cells (John et al., 2013; Rupp et al., 2017; Choi et al., 2019; Hu et al., 2019). Besides, constructing CAR-T cells that can produce PD-1-neutralizing proteins is also in action (Li et al., 2017; Rafiq et al., 2018; Yin et al., 2018; Nakajima et al., 2019). In this study, we constructed mesothelin-specific CAR-T cells with optimized secretion of α-PD-1 scFv and checked the anti-tumor efficacy of CAR-T cells with α-PD-1 scFv *in vitro* and *in vivo*.

## Materials and Methods

### Cell Lines and Culture

Human lung cancer cell line H322 and human embryonic kidney cell line 293T were purchased from the Cell Bank of Chinese Academy of Sciences (Shanghai, China). H322 and 293T cells were cultured in DMEM media (Gibco) with 5% fetal bovine serum (FBS), 100 IU/ml penicillin and 100 μg/ml streptomycin (Invitrogen), supplemented with anti-mycoplasma reagent (Invivogen).

### Vector Construction

α-PD-1 scFv was derived from Nivolumab, whose sequence was obtained from IMGT^1^. Together with the signal peptides (Guler-Gane et al., 2016), the sequences coding α-PD-1 scFv were synthesized by Sangon Inc., and then inserted into lentivirus vector pCDH-EF1 (Systembio). Likely, the sequence of mesothelin-specific 4-1BB-containing CAR was synthesized according to previous report (Carpenito et al., 2009). And P2A linker was used to secure the co-expression of CAR and scFv. Those CAR coding sequences were also inserted into pCDH-EF1 lentiviral vector.

### Lentivirus Production and T Cell Transduction

The modifications of primary T cells with lentivirus were performed as reported (Shi et al., 2019). Briefly, lentivirus was produced in 293T cells by calcium phosphate-DNA co-precipitation. CD3+ T cells were magnetically purified from the peripheral blood mononuclear cells (PBMCs) of health donors and then activated with anti-CD3/CD28 Dynabeads (Thermo Fisher, Cat. No. 111.32D) for 48 h. Spinfection was used to enhance the transduction efficacies of T cells by lentivirus. After infection, T cells were cultured in RPMI1640 media supplemented with 5% FBS and IL-2 (200 IU/). Every 2 or 3 days, fresh media were added and T cells were expanded.

### Detection of T Cell Proliferation and Activation

T cells were labeled with CFSE (Sigma, Cat. No. 21888) and activated by anti-CD3/CD28 Dynabeads for 24 h. Then the activated T cells were mixed with PD-L1-overexpressing H322 cells, which had been fixed using paraformaldehyde (Sigma Aldrich), at the ratio of 1:1 and transferred into 0.4 μm transwell chambers. The chambers were placed in plates, in which 293T cells transiently transfected with α-PD-1-coding vector or mock vector were seeded at the bottoms in advance. After another 24 h, cells in the chambers were collected, stained with fluorochrome-conjugated anti-CD3 (Biolegend, Cat. No. 300318) and anti-CD107a (Biolegend, Cat. No. 328608) antibodies, fixed with paraformaldehyde, and then stained with fluorochrome-conjugated Ki67- and IFN-γ-specific antibodies (Biolegend, Cat. Nos. 652406 and 502528). Then samples were subjected to FACS analysis and the CFSE dilution and target genes expressions were determined in CD3^+^ cells.

### Cytotoxicity Assay

H322 cells were infected with lentivirus expression FFluc-T2A-Puro and purified. CAR-T cells were co-cultured with FFluc-expressing H322 cells at various Effector: T cell (E:T) ratios in RPMI1640 media supplemented with 5% FBS for 18 h, without exogenous cytokines. Then cells were centrifuged, washed and detected using IVIS Imager (Perkin Elmer) after adding 150 μg/ml D-luciferin (Gold Biotechnology, Cat. No. LUCNA-2G) in RPMI1640 media for 5 min. The relative viabilities of tumor cells to those at E:T = 0:1 were calculated.

### Enzyme Linked Immunosorbent Assay (ELISA)

Chimeric antigen receptor T cells were co-cultured with H322 cells at 1:1 for 24 h without exogenous cytokines. Then the supernatant were tested for IL2 and IFN-γ with cytokine-specific ELISA kits (Biolegend, Cat. Nos. 431804 and 430101) according to the instructions.

### Cell Immunofluorescence

PD-1-overexpressing (PD-1+) or –negative (PD-1-) cells were adherent overnight. Then the supernatants of 293T-cells transfected with α-PD-1-expressing vectors were added. 12 h later cells with different PD-1 expressions were washed with phosphate buffered saline (PBS) for three times and fixed with 4% paraformaldehyde. The fixed cells were incubated with Alexa Fluor^®^ 488 His tag-specific antibody (Thermo Fisher, Cat. No. MA1-21315-A488) at 4°C overnight. After washing, cells were stained with 0.1 μg/ml DAPI (Thermo Fisher, Cat. No. 62248). The stained cells were observed under fluorescence microscope (Leica, Germany), and photos were recorded.

### Western Blot

After 48 h transfection, the supernatants and 293T cells were collected, respectively. The supernatants were dripped to NC membranes (GE Healthcare Bio-Sciences). Blocked by 5% skim milk, NC membranes were sequentially incubated with primary antibody to His tag (Cell signaling technology, Cat. No. 2366S) for 2 h and HRP-conjunct secondary antibody (Cell signaling technology, Cat. No. 7076P2) for 1 h at room temperature. The dots were developed using Enhanced Chemiluminescence Solution (Thermo Fisher, Cat. No. 32109).

The detection of whole cell lysates were carried out as described in previous report (Song et al., 2019). Briefly, 293T cells were fractured using lysis buffer containing RIPA (Beyotime, Cat. No. P0013B), protease inhibitors (Sigma Aldrich, Cat. No. P8340), phosphatase inhibitors (Sigma Aldrich, Cat. No. P5726) and PMSF (Solarbio, Cat. No. P0100). After ultracentrifuge, the lysates were loaded into 10% SDS-PAGE gels and proteins were isolated. Then proteins were transferred to NC membrane. Membranes were stained with primary antibodies specific for His tag and β-actin. After incubation with HRP-conjunct secondary antibody, bands were developed.

### Protein-L Staining for FACS Detection

To detect the surface expression of CAR, Protein-L staining was carried out as reported (Zheng et al., 2012). Briefly, 1 × 10^6^ CAR-T cells were suspended in 0.2 mL ice-cold wash buffer (PBS containing 2% BSA) with 0.02 μg protein L (GenScript, Cat. No. M00097) for 30 min. After washing three times, CAR-T cells were incubated with 1 μL fluorochrome-conjugated streptavidin (Biolegend, Cat. No. 405203) for 30 min. Then CAR-T cells were washed two times and detected with FACS Canto II cytometer (BD bioscience, United States).

### Imaging Flow Cytometry

Chimeric antigen receptor T cells activated by anti-CD3/CD28 Dynabeads were harvested and washed two times. Fluorochrome-conjugated anti-human PD-1 (Biolegend, Cat. No. 329903) and anti-His tag antibodies were co-incubated with CAR-T cells at 4°C for 15 min. Then T cells were counter-stained with DAPI. Then T cells were detected using ImageStream MKII system (Amnis, Germany).

### Immunohistochemistry (IHC)

Mice lungs with tumor lesions were fixed, embedded in paraffin and serially sliced. Paraffin-embedded tissue slides were dewaxed in 65°C for 1 or 2 h, hydrated in alcohol with different concentrations, heated in citrate buffer for antigen retrieval, and incubated with hydrogen peroxide for 15 min to inactivate endogenous peroxidases. Anti-human CD3 antibody (Cell signaling technology, Cat. No. 85061T) or primary antibody to His tag was added on slides and incubated at 4°C overnight. On the next day, slides were stained with HRP-conjunct anti-rabbit antibody (Cell Signaling Technology, Cat. No. 7074S or 7076P2). CD3 expressions were visualized by DAB staining (Dako, Cat. No. K3468) following slides being counterstained with hematoxylin. The photos were recorded by inverted microscope (Leica, Germany).

### Xenograft Tumor Model

Female SCID-Beige mice aged at 5–6 weeks were obtained from Vital River (Beijing, China). For each mouse, 1 × 10^6^ H322 cells expressing luciferase were inoculated subcutaneously. When the mean total flux of tumors reached 1 × 10^9^ p/s, mice were intravenously infused with 5 × 10^6^ CAR-meso cells, CAR-meso-α-PD-1 cells and equal volumes of PBS, respectively. Tumor growth was checked weekly. To monitor tumor growth, mice were anesthetize and intraperitoneally injected with 3 mg D-luciferin. After 10 min, mice were imaged using IVIS Imager.

### FACS Analysis of Tumor-Infiltrating T Cells

Five days after CAR-T cells injection, the subcutaneous tumors were isolated and dispersed. The single cell suspensions were washed with PBS containing 2% FBS and stained with Fixable Viability Dye (Thermo Fisher) and fluorochrome-conjugated antibodies specific to human CD45 (Biolegend, Cat. No. 304014), CD3 (Biolegend, Cat. No. 300406), CD69 (Biolegend, Cat. No. 310910), CD27 (Biolegend, Cat. No. 302838), PD-1 (Biolegend, Cat. No. 329906), Tim3 (Biolegend, Cat. No. 345006), CTLA4 (Biolegend, Cat. No. 369612), IFN-γ (Biolegend, Cat. No. 502528), and Granzyme B (Biolegend, Cat. No. 515406) at 4°C for 15 min. Cells were detected on FACS Canto II cytometer (BD bioscience, United States). The data were further analyzed using FlowJo version 10 (Becton, Dickinson and Company).

### Orthotopic Tumor Model

Female SCID-Beige mice aged at 5–6 weeks were injected with 5 × 10^5^ luciferase-expressing H322 cells intravenously. One week later, mice were infused through tail veins with 5 × 10^6^ CAR-meso cells, CAR-meso-α-PD-1 cells and equal volumes of PBS, respectively. To monitor tumor growths, each mouse was intraperitoneally injected with 3 mg D-luciferin weekly, and mice were imaged using IVIS Imager after 10 min.

### Statistical Analysis

Data presented as mean ± SEM were representative of at least three independent repeats. *T*-test and ANOVA comparison were performed to test the differences among groups. Survivals were depicted using Kaplan-Meier curves and analyzed by log-rank test. *P* < 0.05 was recognized as statistically significant. Statistical analyses were performed in Prism Version 7 (GraphPad).

## Results

### Construction of the Secretory α-PD-1 scFv

**FIGURE 1:**
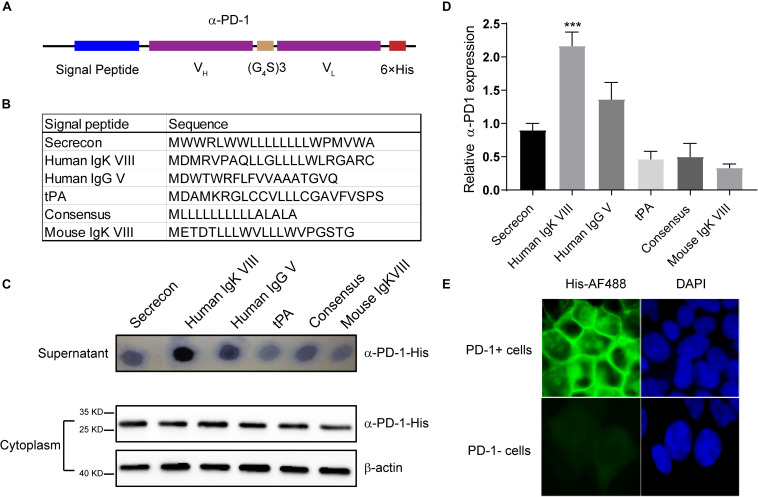
Characterization of self-delivered α-PD-1 scFv. **(A)** Schematic structure of secretory α-PD-1 scFv. **(B)** Sequences of signal peptides tested. **(C)** 293T cells were transfected with vectors coding His-tagged α-PD-1 scFv with different leading signals. 48 h later, the supernatants and 293T cells were separately collected and subjected to western blot analysis. **(D)** The expressions of interested proteins in the supernatants were measured according to gray-values using ImageJ software. Then relative expressions to Secrecon were calculated. **(E)** PD-1+ or PD-1- 293T cells were incubated with the supernatants from 293T cells expressing α-PD-1 scFv. Then the binding of scFv to cells having different PD-1 expressions were detected with AF488-labeled His tag-specific antibody. Data shown were representative of three independent experiments. *** indicates *P* < 0.001.

Based on the sequence of Nivolumab obtained from IMGT, we designed the secreted scFv with His tag (Figure 1A). To obtain the optimal secretion of the scFv, we compared 6 signal peptides frequently used in engineering secretory proteins (Guler-Gane et al., 2016; Figure 1B). As shown in Figure 1C, the leading peptide originated from human IgK VIII resulted in enhanced extracellular accumulations of anti-PD-1 scFv, although all constructs with different signal peptides were similarly produced in cells. Statistical analysis showed that the secreting capacity of anti-PD-1 scFv was obviously enhanced by human IgK VIII signal domain (*P* < 0.01) (Figure 1D). To confirm whether the secreted PD-1-neutralizing scFv could bind with the target protein, we added the supernatants containing anti-PD-1 scFv into PD-1-positive or -negative 293T cells. Immunofluorescence analysis demonstrated that human IgK VIII signal peptide-containing α-PD-1 scFv could specifically bind with PD-1 (Figure 1E). Therefore, the construct with human IgK VIII leading segment was used in following experiments.

### Secreted α-PD-1 scFv Enhances T Cell Function

**FIGURE 2:**
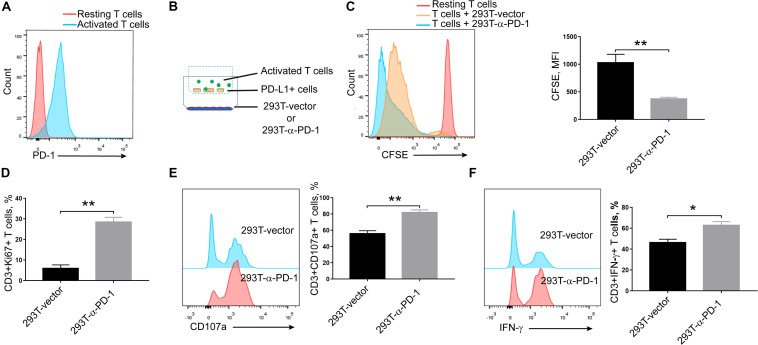
Effects of secreted α-PD-1 scFv on T cell activation. CD3+ T cells from healthy donors were stained with CFSE and then activated by anti-CD3/CD28 beads for 2 days. **(A)** PD-1 expressions were determined in resting and activated T cells by FACS assay. **(B)** 293T cells transfected with mock or α-PD-1 scFv-expressing vectors were plated at the bottom. 48 h later, activated T cells were added into the upper transwell chambers with fixed PD-L1-overexpressing A549 cells at the ratio of 1:1 for 24 h. **(C)** Then the dilutions of CFSE in CD3+ T cells were determined using FACS assay. **(D–F)** The expressions of Ki67 **(D)**, CD107a **(E)** and IFN-γ **(F)** were further detected in CD3+ T cells after incubation. T cells from 5 donors were tested and the representative results were depicted. * indicates *P* < 0.05; ** indicates *P* < 0.01.

Seeing that α-PD-1 scFv was efficiently secreted and specifically bound to the inhibitory PD-1 receptor, we then checked whether the secreted proteins maintained the neutralizing effects. As shown in Figure 2A, PD-1 was robustly induced in activated T cells. Then the activated T cells and PD-L1-overexpressing A549 cells were added into upper chambers within culture plates, in which anti-PD-1 scFv-producing or mock cells had been seeded in advance (Figure 2B). As expected, the supernatants from anti-PD-1 scFv-producing cells but not the mock cells enhanced the proliferations of T cells (*P* < 0.01) (Figure 2C). Consistently, Ki67 expressions were enhanced in T cells when anti-PD-1 scFv was present (*P* < 0.01) (Figure 2D). In addition, CD107a and intracellular IFN-γ were higher in T cells co-culture with anti-PD-1 scFv-producing 293T cells than that co-incubated with mock cells (*P* < 0.05) (Figures 2E,F), indicating the secreted scFv alleviates the inhibitory effects of PD-1.

### Characterization of CAR-T Cells Secreting α-PD-1 scFv

**FIGURE 3:**
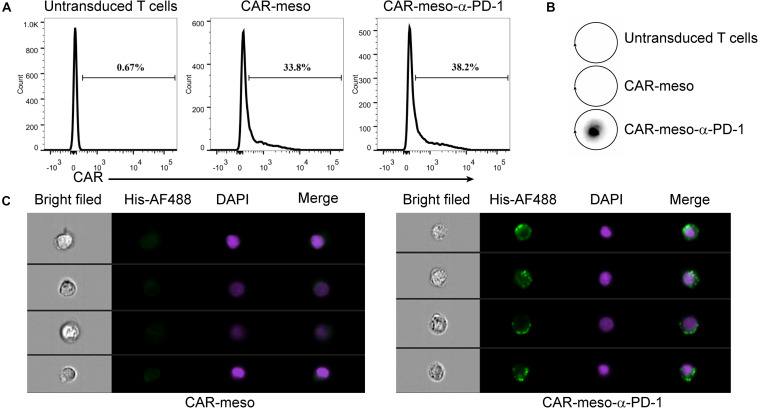
Construction of CAR-T cells secreting α-PD-1 scFv. **(A)** The surface expressions of CAR were determined by protein-L staining using FACS test. **(B,C)** The secreted α-PD-1 scFv with His tag by CAR-T cells cold bind with CAR-T cells. CAR-T cells were activated by anti-CD3/CD28 beads for 24 h. Then the supernatants were subjected to western blot assay and scFv secretion from CAR-meso-α-PD-1 cells were confirmed **(B)**. CAR-meso and CAR-meso-α-PD-1 cells were further stained with AF488-labeled antibody against His tag, and checked on image flow cytometer **(C)**. CAR-T cells derived from 5 different donors were tested and the representative results were shown.

Then we constructed mesothelin-specific CAR-T cells that could secret α-PD-1 scFv (CAR-meso-α-PD-1) or not (CAR-meso). As shown in Figure 3A, the transduction efficacies of primary T cells were not affected by the addition of α-PD-1 scFv and CAR-T cells were able to secret the α-PD-1 scFv (Figure 3B). Furthermore, the secreted scFv efficiently bound with activated T cells, which expressed high levels of PD-1 (Figure 3C).

### Self-Delivered α-PD-1 scFv Enhances CAR-T Cell Functions

**FIGURE 4:**
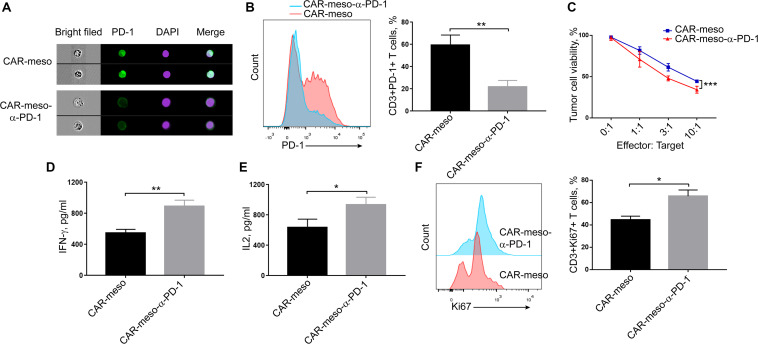
Blockade of PD-1 suppression by self-delivered α-PD-1 scFv. **(A,B)** Activated CAR-T cells were stained with anti-PD-1 antibody and checked on image flow cytometer **(A)** and the expressing levels of PD-1 were determined **(B)**. **(C–E)** To check the effects of scFv on the cytotoxic activities, CAR-T cells were directly incubated with H322 cells without pre-activation by anti-CD3/CD28 Dynabeads. CAR-T cells were co-cultured with luciferase-expressing H322 cells at indicated E:T ratios for 18 h. Then the relative viabilities of tumor cells were determined and compared between treatments with CAR-meso or CAR-meso-α-PD-1 cells **(C)**. Additionally, CAR-T cells were co-incubated with H322 cells at the ratio of 1:1 for 24 h. Then the supernatants were checked for the secretions of IFN-γ **(D)** and IL2 **(E)**. The cells were collected and Ki67 expressions were determined in CD3+ T cells using FACS test **(F)**. CAR-T cells derived from 5 different donors were tested and the representative data were demonstrated. * indicates *P* < 0.05; ** indicates *P* < 0.01.

As seen in Figure 4A, the bound scFv obviously blocked PD-1 on activated T cells. Compared with CAR-meso cells, PD-1 was significantly decreased on CAR-meso-α-PD-1 cells (*P* < 0.005) (Figure 4B). Then we tested whether the secreted anti-PD-1 scFv could enhance CAR-T cell functionality when then engineered T cells were encountered with mesothelin-positive H322 cells. As expected, the scFv enhanced the cytotoxicity of CAR-T cells against tumor cells (Figure 4C). Similarly, the secretions of effector cytokines, IFN-γ and IL-2 were upregulated in CAR-T cells expressing PD-1 neutralizing scFv (*P* < 0.05) (Figures 4D,E). As well, the proliferations of CAR-T cells were enhanced by secreted PD-1-neutralizing scFv (*P* < 0.05) (Figure 4F). Those observations suggested that the tandem design of CAR with anti-PD-1 scFv improves the functionalities of CAR-T cells by blocking the inhibitory effects of PD-1.

### CAR-T Cells Secreting α-PD-1 scFv Demonstrate Improved Anti-tumor Effectiveness *in vivo*

**FIGURE 5:**
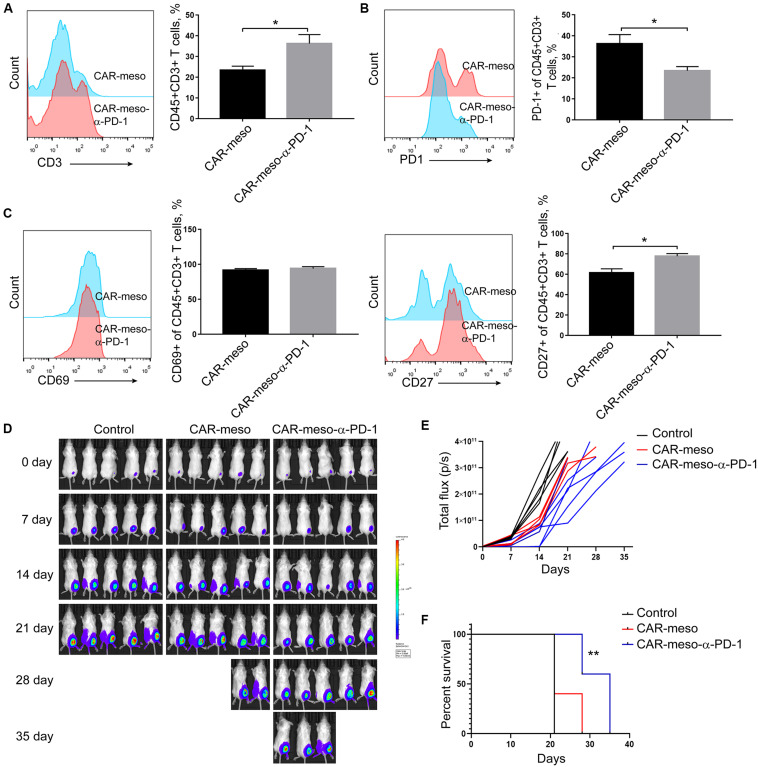
Enhanced anti-tumor capacity of CAR-meso-α-PD-1 cells. Luciferase-expressing H322 cells were inoculated into SCID-Beige mice subcutaneously. When the tumor volumes were around 80 mm^3^, mice were treated with CAR-meso cells, CAR-meso-α-PD-1 cells and PBS, respectively. Before infusion, CAR-T cells had been purified and the purities were over 95%. **(A–C)** 5 days after treatment, tumors were isolated and the single cell suspensions were prepared (*n* = 4 in each group). After excluding dead cells with fixable viability dye, the accumulations of CAR-T cells within malignant tissues were determined according to CD45 and CD3 staining **(A)**. Furthermore, PD-1 **(B)**, CD69 and CD27 **(C)** were detected in CD45+CD3+ cells. **(D,E)** Tumor volumes were recorded weekly by IVIS imager (*n* = 5 per group). **(F)** Survivals of mice receiving indicated treatments. * indicates *P* < 0.05; ** indicates *P* < 0.01.

To further investigate the effects of anti-PD-1 scFv, we tested the anti-tumor effects of CAR-T cells in H322-xenografted mouse models. When the subcutaneous tumors were established, CAR-meso or CAR-meso-α-PD-1 cells were infused through tail veins. Five days later, tumor-infiltrating CAR-T cells were isolated and subjected to FACS assay. As expected, PD-1 in CAR-T cells was blocked by the secreted α-PD-1 scFv (*P* < 0.05) (Figure 5B). Correspondingly, the accumulations (*P* < 0.05) (Figure 5A), activations (*P*_CD27_ < 0.05) (Figure 5C) and the release of cytotoxic cytokines (*P*_IFN–γ_ < 0.0004, *P*_GranzymeB_ < 0.0019) (Supplementary Figures S1A,B) within tumor tissues of CAR-T cells were enhanced by the self-delivered α-PD-1 scFv. With regarding to the expression of Tim3 and CTLA4, there was mild difference between CAR-meso and CAR-meso-α-PD-1 cells within tumors (Supplementary Figures S1C,D). Then we checked the anti-tumor effects of CAR-T cells. Compared with PBS-treated group, CAR-meso cells delayed tumor growth (Figures 5D,E). Nevertheless, CAR-meso cells could not obviously improve the survival of mice bearing the established tumors, which were probably highly suppressive (Figure 5F). In contrast, CAR-meso-α-PD-1 cells not only inhibit tumor growth (Figures 5D,E) but also extended the survival (*P* < 0.05) (Figure 5F). In addition, we also detected the α-PD-1 scFv secretion in different tissues and found that α-PD-1 scFv was majorly accumulated in tumor tissues (Supplementary Figure S2). Those observations indicated that the constructed α-PD-1 scFv-secreting CAR-T cells have more potent anti-tumor effects in the immune-suppressive niche.

### CAR-Meso-α-PD-1 Increase T Cells Infiltration in Tumor Microenvironment

**FIGURE 6:**
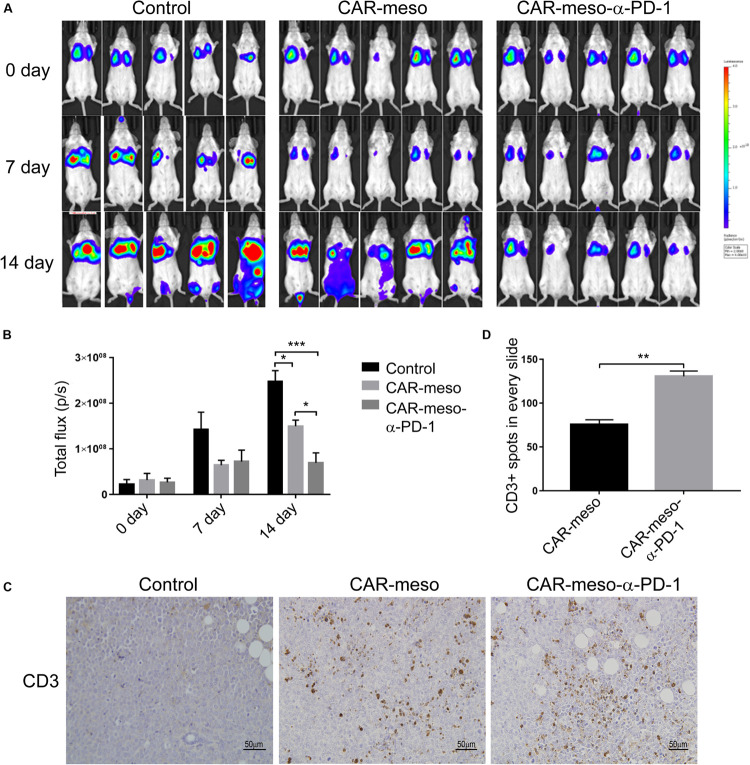
Tumor-killing potency of CAR-T cells against orthotopic tumors. Orthotopic tumors were established by injecting luciferase-expressing H322 cells through tail veins. Then mice were given the listed treatments (*n* = 5 per treatment). **(A,B)** Tumor volume were determined according IVIS image weekly **(A)** and compared as indicated **(B)**. **(C,D)** On the 14th day post treatment, the infiltrations into tumors of CAR-T cells were checked by IHC technique **(C)**. And the numbers of CD3+ cells were counted and statistically compared **(D)**. * indicates *P* < 0.05; ** indicates *P* < 0.01; *** indicates *P* < 0.001.

We also detected the tumoricidal efficacies of CAR-T cells in orthotopic tumor models. As shown in Figure 6A, CAR-T cells efficiently suppressed the progression of tumors in comparison with PBS treatment. And the α-PD-1 scFv-secreting CAR-T cells demonstrated more potent effectiveness than CAR-meso cells (*P* < 0.05) (Figures 6A,B). Further analysis showed that both types of CAR-T cells infiltrated into the malignant tissues (Figure 6C). But the enhanced proliferations and accumulations of T cells were noticed in mice treated with CAR-meso-α-PD-1 cells (*P* < 0.01) (Figures 6C,D), which had demonstrated better anti-tumor effects (Figures 6A,B). Together with above data, it was inferred that the self-delivered PD-1-neutralizing scFv can reduce the suppressive effects of PD-1 and enhance CAR-T cell activity within solid tumors.

## Discussion

Chimeric antigen receptor T cells can infiltrate into tumors and restrain the progression of malignant tissues (Du et al., 2019; Lv et al., 2019). Nevertheless, the potency of CAR-T cells are largely restricted within solid tumors due to the immune-suppression (Gargett et al., 2016; Mardiana et al., 2019). Upon activation, PD-1 is robustly induced in CAR-T cells and suppressed the cytotoxic functions of CAR-T cells when this inhibitory receptor binds with PD-L1, which is abundant in the microenvironment (John et al., 2013; Moon et al., 2014; Cherkassky et al., 2016; Zolov et al., 2018; Shi et al., 2019). Several studies have reported that interfering PD-1 activation is an effective approach to enhance CAR-T cells in solid tumor (Cherkassky et al., 2016; Chong et al., 2017; Li et al., 2017; Serganova et al., 2017). In this study, we explored whether the self-delivered short α-PD-1 scFv could enhance CAR-T cell efficacy.

RNAi and CRISPR/cas9 have been used to downregulate PD-1 expression. CAR-T cells electroporated with PD-1-specific siRNA demonstrate increased anti-tumor efficiency when incubated with PD-L1-overexpressed melanoma cells (Simon et al., 2018). Similarly, downregulation of PD-1 by CRISPR/Cas9 relieves immune-inhibition and enhances CAR-T cell function in solid tumors (Rupp et al., 2017; Hu et al., 2019). However, RNAi- or CRISPR/Cas9-mediated PD-1 reduction are restricted in CAR-T cells themselves. The short persistence of siRNA and low efficacies of gene editing may further limit such approaches to produce upgraded CAR-T cells that uniformly have long-lasting tumoricidal effects. A better strategy to augment anti-tumor efficacy is to block PD-1 activation with neutralizing proteins within microenvironment, which are able to increase the activations of not only the engineered CAR-T cells but also the surrounding CAR-T cells and natural tumor-responsive T cells.

Previous studies have shown that the neutralizing antibody specific for PD-1 can improve CAR-T cell activities within tumors and enhance the therapeutic effectiveness (John et al., 2013; Zhang et al., 2017). However, there are some side-effects for the systemic infusion of PD-1-blocking antibody, such as dermatitis, colitis or hepatitis, due to the activated T lymphocyte-induced immune-related adverse events (Hofmann et al., 2016; Shivaji et al., 2019). Alternatively, it is feasible to overcome the obstacles by constructing CAR-T cells that can secret the PD-1-neutralizing proteins. α-PD-1 antibody Nivolumab is widely used in clinic (Baxi et al., 2018). Here, we tested CAR-T cells producing PD-1-blocking peptides based on the scFv version of Nivolumab. To obtain optimal blockade, we first tested the signal peptides. Signal peptides, consisted of several amino acids, play determinant roles in protein distribution (Mori et al., 2015; Owji et al., 2018; Almagro Armenteros et al., 2019). Among the signal peptides tested, it was noticed that human IgK VIII enhanced the extracellular transportation of α-PD-1 scFv (Figure 1). The produced scFv with human IgK VIII leading signal could efficiently associate with PD-1 (Figure 1) and enhance T cell activation (Figure 2). CAR-T cells producing the scFv also demonstrated enhanced cytotoxicity against solid tumor *in vitro* and *in vivo* (Figures 4–6). Especially, when the antitumor functions of CAR-T cells were largely compromised in the advanced tumors having strong immune-suppression, the anti-PD-1 scFv-producing cells demonstrated enhanced cytotoxicity and delayed tumor growth (Figure 5). Consistently, the self-delivery of PD-1-neutralizing scFv have been tested in CAR-T cells (Li et al., 2017; Rafiq et al., 2018). It is safe and potent in treating solid tumors with CAR-T cells that can produce the secretory α-PD-1 peptides. However, those studies have not explored the impacts of leading signals on the secretions of scFv (Li et al., 2017; Rafiq et al., 2018). Our worked demonstrated that the extracellular accumulations of PD-1 blocking scFv were affected by the leading signals (Figure 1). The differences in scFv secretion may eventually bring on different clinical outcomes. More detailed explorations are needed to determine the optimal format of self-delivered α-PD-1 scFv in CAR-T cells.

PD-1-mediated immune suppression of CAR-T cells is a huge challenge in treatment of solid tumors. The combination with PD-1 blockade can efficiently improve the activities of CAR-T cells in solid tumors. Our work showed that CAR-T cells could act as the producer of α-PD-1 scFv and enhance their activities by themselves in solid tumors. The α-PD-1 scFv-secreting CAR-T cells represent a possible solution for effective engineered T cell therapy of solid tumors.

## Data Availability Statement

The raw data supporting the conclusions of this article will be made available by the authors, without undue reservation.

## Ethics Statement

The studies involving human participants were reviewed and approved by the Ethics Committee of the First Affiliated Hospital of Zhengzhou University. The patients/participants provided their written informed consent to participate in this study. The animal study was reviewed and approved by the Ethics Committee of the First Affiliated Hospital of Zhengzhou University.

## Author Contributions

YZ designed this study. YP, FL, and SN performed the experiments. DZ, XS, and JS helped to complete the experiment. All authors contributed to the article and approved the submitted version.

## Conflict of Interest

The authors declare that the research was conducted in the absence of any commercial or financial relationships that could be construed as a potential conflict of interest.

## Footnotes

1 http://www.imgt.org/

## Supplementary Material

FIGURE S1 Exhaustion makers and cytotoxic molecules analysis of CAR-meso and CAR-meso-α-PD-1 scFv cells. Quantification of IFN-γ **(A)**, Granzyme B **(B)**, Tim3 **(C)** and CTLA4 **(D)** detection by flow cytometry on CAR-meso and CAR-meso-α-PD-1 scFv cells T cells. Data shown is mean ± SEM. ** indicates *P* < 0.01; *** indicates *P* < 0.001.

FIGURE S2 Validation of α-PD-1 scFv secretion in various tissues. His tag was used in IHC experiment for α-PD-1 scFv detection in lung, liver, spleen, and tumor tissues.
