# Supplementary figures and images for: Augmenting the Effectiveness of CAR-T Cells by Enhanced Self-Delivery of PD-1-Neutralizing scFv
Source: Front Cell Dev Biol. 2020 Aug 18;8:803. doi: 10.3389/fcell.2020.00803 (PMC7461868; doi:10.3389/fcell.2020.00803)

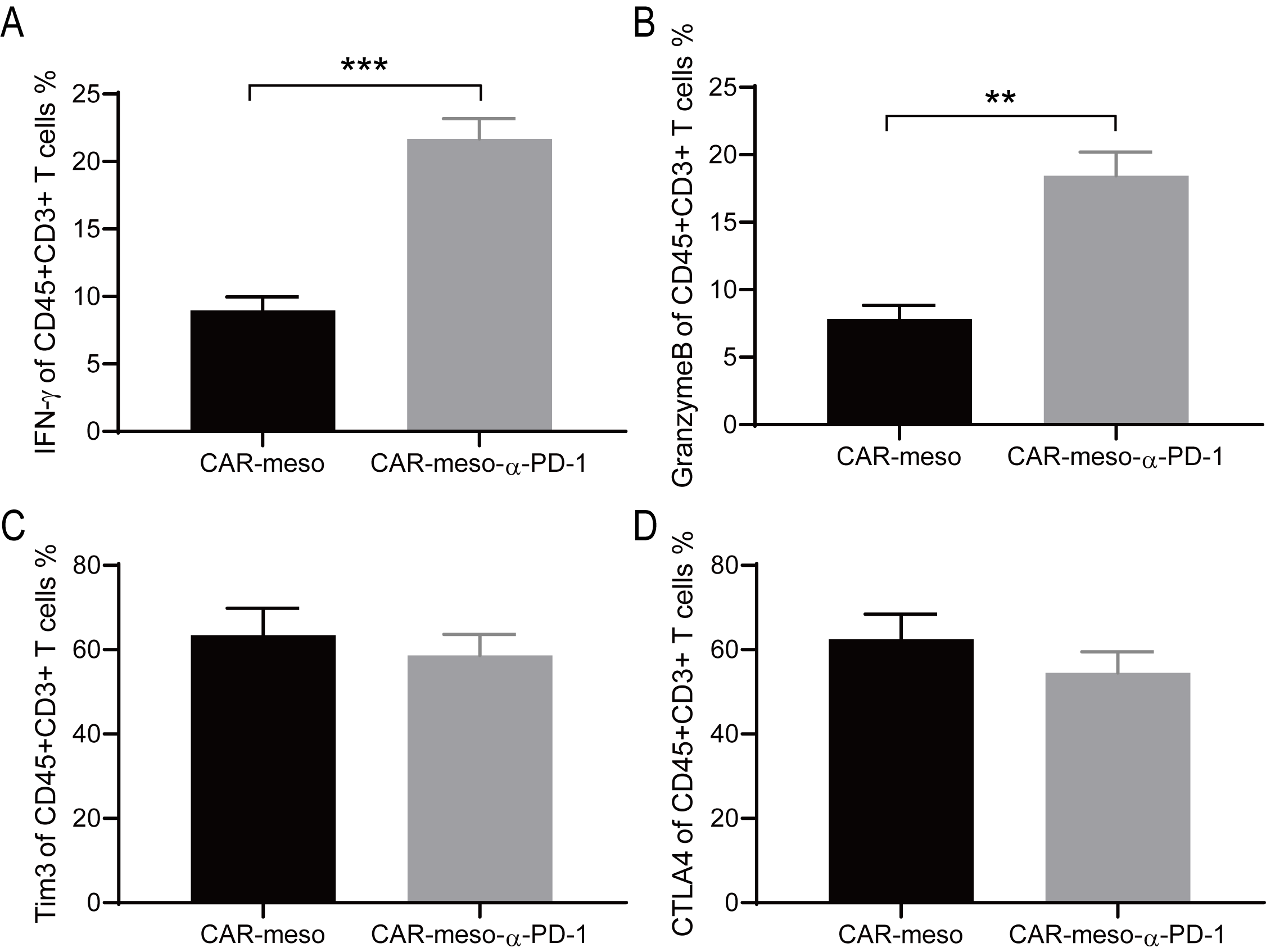

Supplement: FIGURE S1 — Exhaustion makers and cytotoxic molecules analysis of CAR-meso and CAR-meso-α-PD-1 scFv cells. Quantification of IFN-γ (A), Granzyme B (B), Tim3 (C) and CTLA4 (D) detection by flow cytometry on CAR-meso and CAR-meso-α-PD-1 scFv cells T cells. Data shown is mean ± SEM. ** indicates P < 0.01; *** indicates P < 0.001. [file Image_1.tif]

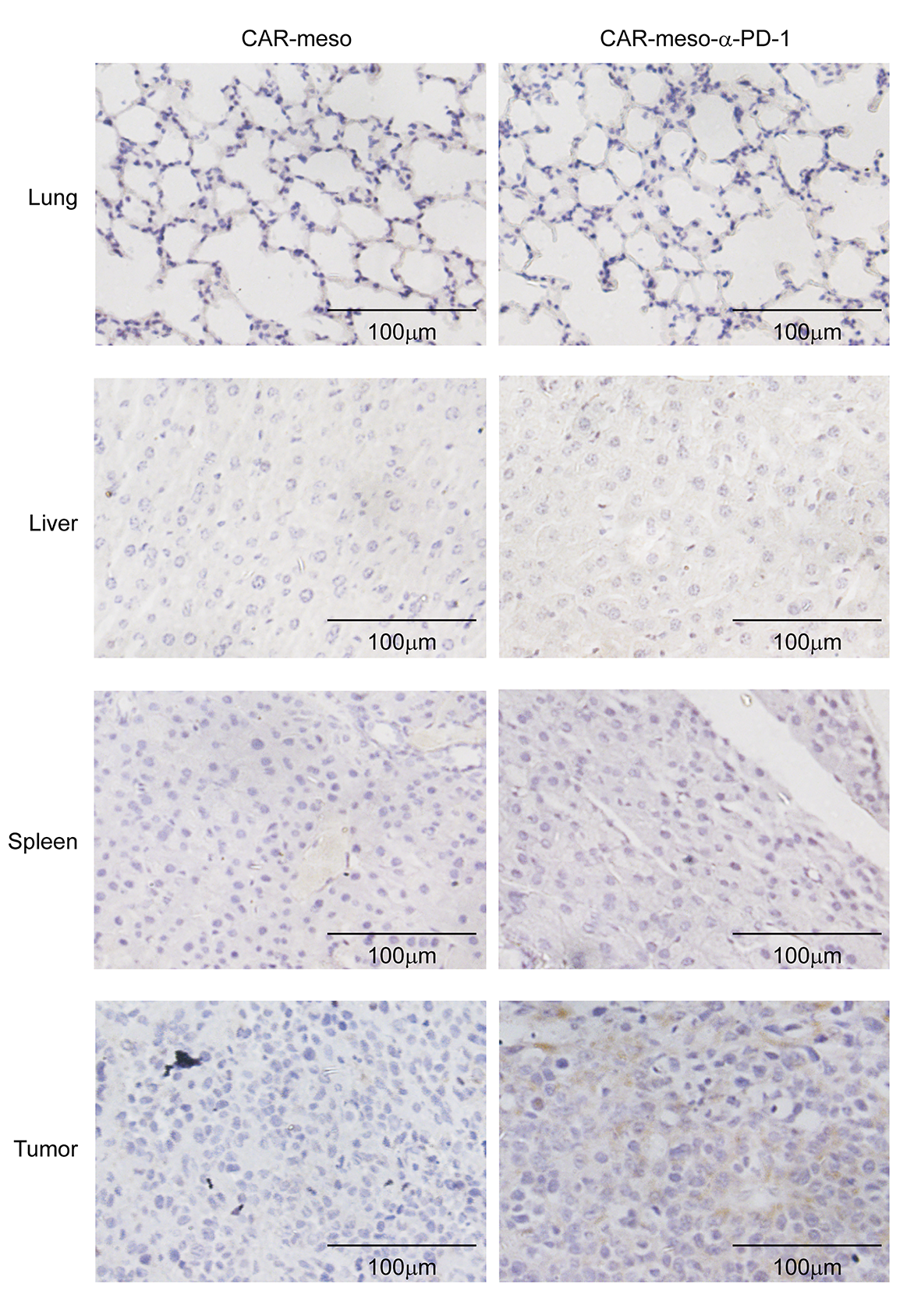

Supplement: FIGURE S2 — Validation of α-PD-1 scFv secretion in various tissues. His tag was used in IHC experiment for α-PD-1 scFv detection in lung, liver, spleen, and tumor tissues. [file Image_2.tif]
